# Challenges of liver transplantation programs in low‐ and middle‐income countries: An experience from Sri Lanka

**DOI:** 10.1002/puh2.162

**Published:** 2024-02-20

**Authors:** M. J. S. Jayarathna, B. K. Dassanayake, Thinley Dorji, Don Eliseo Lucero‐Prisno, S. Samarasinghe, Vasanthi Pinto, M. D. Lamawansa

**Affiliations:** ^1^ Department of Anatomy Faculty of Medicine University of Peradeniya Kandy Sri Lanka; ^2^ Department of Surgery Faculty of Medicine University of Peradeniya Kandy Sri Lanka; ^3^ Teaching Hospital Peradeniya Kandy Sri Lanka; ^4^ Department of Internal Medicine Central Regional Referral Hospital Gelephu Bhutan; ^5^ Department of Global Health and Development London School of Hygiene and Tropical Medicine London UK; ^6^ Department of Anaesthesiology and Critical Care Faculty of Medicine University of Peradeniya Kandy Sri Lanka

**Keywords:** end‐stage liver disease, liver cirrhosis, liver transplantation, organ transplantation, Sri Lanka

## Abstract

Liver diseases lead to 1.3 million deaths per year around the world, the majority of which are secondary to cirrhosis. In the management of liver diseases in chronic and acute conditions, liver transplant (LT) plays a major role in improving the survival and quality of life of patients. LT programmes require the technical capabilities in performing the pre‐transplant evaluation, transplant surgery and post‐transplant care supported by adequate infrastructure and a set of trained teams. Globally, there were 28,000 deceased donor LTs, and 14,000 living donor LTs were performed in 2021. In the South Asia region, India, Pakistan and Sri Lanka conducted 2998 LTs in 2021. Many countries report sociocultural, religious and legislative barriers to acquiring adequate donor livers. We describe the challenges in LT programmes in low‐ and middle‐income countries and experiences from Sri Lanka. Sri Lanka carried out its first LT in 2010, and the service is provided free of charge in the state health sector. In Sri Lanka, the common indications for LT in adults are non‐alcoholic steatohepatitis, cirrhosis, hepatocellular carcinoma and alcoholic liver disease. In children, the indications are biliary atresia, hepatocellular carcinoma and Wilson disease. The common challenges include a lack of an adequate number of doctors and post‐transplant team members, a low number of organ donors and a long waiting list, all of which can be disadvantageous for transplant programmes. To continue providing LT services, there is a need to adopt multimodal strategies in the areas of providing additional skills training to the operating team and promoting organ donation culture in the background of supportive organ donation legislation. With the adoption of the national strategic plan for organ, tissue and cell transplantation, the country hopes to strengthen its capacity of providing transplant services to its people.

## INTRODUCTION

Liver diseases are the 11th leading cause of death worldwide accounting for 1.3 million deaths per year, among which approximately two‐thirds are men [1, 2]. In the South Asia region, liver diseases are ranked the 10th leading cause of mortality with a crude death rate of 18 per 100,000 population per year [3]. Alcohol‐associated liver disease is by far the major cause of liver disease worldwide, where alcohol often exacerbates liver injury caused by other aetiologies. The other common causes of chronic liver disease are non‐alcoholic fatty liver disease (NAFLD), hepatitis B virus infection and hepatitis C virus infection [1]. Other diseases, including autoimmune and hereditary liver disorders such as primary biliary cholangitis, primary sclerosing cholangitis, alpha‐1‐antitrypsin deficiency, Wilson disease and autoimmune hepatitis, account for about 1% of cases [1]. In addition, acute liver failure, though rare, is an important disease entity with very high rates of mortality. In Sri Lanka, alcohol remains a leading cause of liver diseases followed by NAFLD with a relatively lesser burden of chronic viral hepatitis‐related liver disease compared to countries in the region and a very low prevalence of hepatitis B and C [4, 5]. The prevalence of NAFLD is 18% among rural and 32.6% among urban adult population [6, 7]. In the South Asian region, hepatitis B, C and NAFLD play a major role in chronic liver disease with or without associated hepatocellular carcinoma [8].

Globally, the burden of chronic hepatitis B virus infection was about 296 million people in 2019 with an estimated 15 million new infections and 331,000 deaths during the year 2019 [9], whereas the prevalence of chronic hepatitis C virus infection was 1% in 2015 (around 71 million) with around 1.75 million cases detected every year [3]. In many resource‐limited countries, hepatitis B remains undiagnosed for long periods leading to complications such as cirrhosis and hepatocellular carcinoma. With the implementation of prevention strategies for chronic hepatitis B infection, the age‐adjusted death rate from cirrhosis has declined from 21 to 16.5 per 100,000 population from 1990 to 2017 [1]. However, its overall burden is expected to increase by 39% from 2015 to 2030 [9].

NAFLD occurs in non‐alcoholic fatty liver and steatosis with varying degrees of lobular inflammation, fibrosis and cirrhosis. The increasing prevalence of metabolic risk factors, such as obesity, diabetes, alcohol intake and high‐calorie intake, is driving the increase in the prevalence and progression of NAFLD [3]. Preventive measures for viral hepatitis, vaccination and the global drive for health education on sexually‐ and transfusion‐transmitted infections play a major part in reducing the disease burden. In those where liver disease progresses, liver transplant (LT) serves a major role in improving the disease outcome and quality of life in patients [10].

**FIGURE 1:**
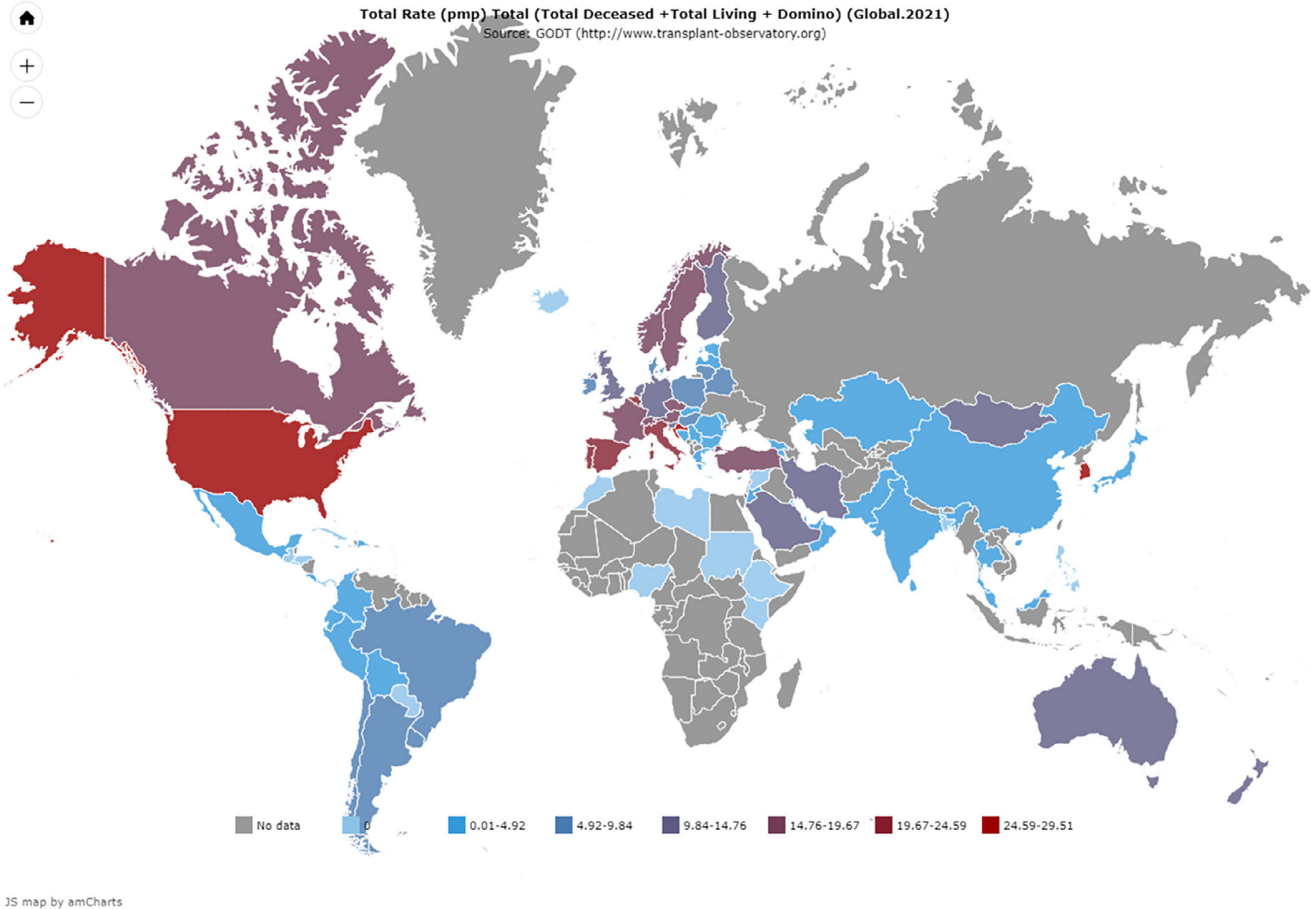
Global volume of organ transplantations performed in 2021 [12].

**FIGURE 2:**
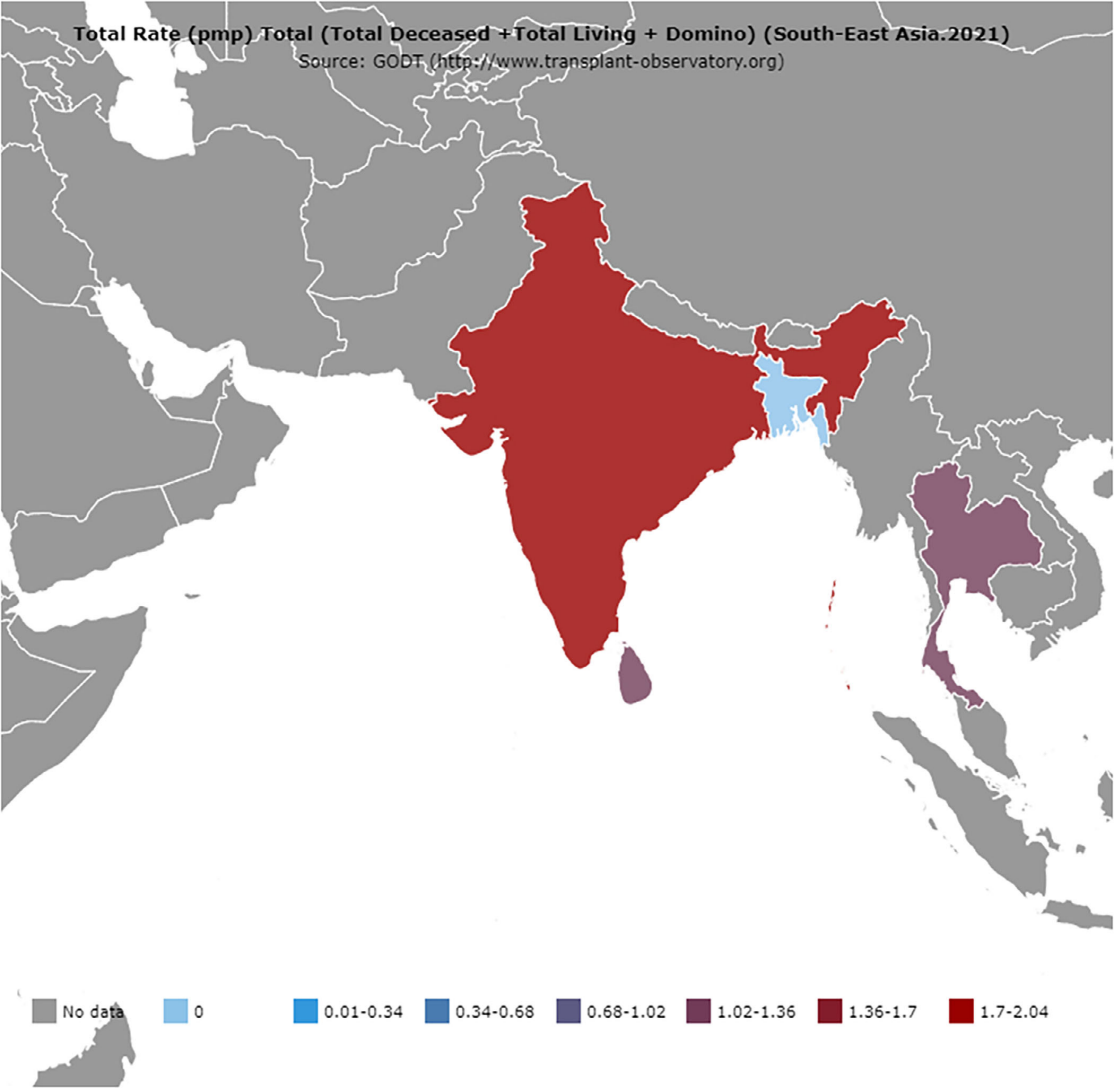
The volume of organ transplantation activities performed in the South–East Asian region in 2021 [12].

**FIGURE 3:**
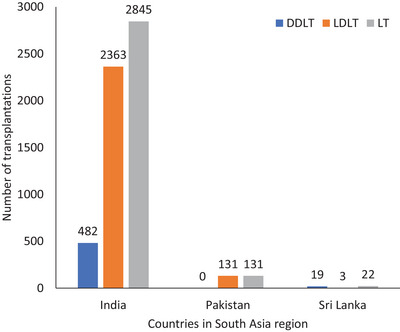
The number of liver transplants performed in South Asia in 2021. DDLT, deceased‐donor liver transplantation; LDLT, living‐donor liver transplantation; LT, liver transplantation [12].

LT surgery has evolved over decades with the involvement of major medical and surgical specialties, including anaesthesia and critical care, haematology and hepatology. The world's first successful human LT surgery was performed in 1963 on a 3‐year‐old child with biliary atresia in the United States [11]. In 2021, around 28,000 deceased donor LTs (DDLTs) and 14,000 living donor LTs (LDLTs) were performed globally, with around 1000 DDLTs and 5000 LDLTs performed in the Southeast Asian region [12]. Figures 1, 2, 3 show the number of liver transplantation surgeries globally and in the South Asian region in 2021 [12]. The top indications for LTs include non‐alcoholic steatohepatitis, hepatocellular carcinoma, alcohol‐associated liver disease and viral hepatitis (B and C) [10, 11, 13].

The development of liver transplantation services requires technical skills, a proper legal framework for organ transplantation and a viable means to support the transplant programme. In many low‐ and middle‐income countries (LMICs) that lack adequate funding for the health sector, organ transplantations are often viewed as high‐end services by the governments as well as hospitals. In this article, we review the scenario of liver diseases and liver transplantation in LMICs with a special focus on describing the experiences from Sri Lanka.

## THE SCOPE OF LIVER TRANSPLANTATION

The surgical techniques and the processes involved in liver transplantation have undergone major improvement with an increase in the 5‐year survival rates from <40% to now 80% and a marked improvement in the quality of life of recipients [11]. South Korea and China are leading countries for live‐donor liver transplantation (LDLTs) performed at comparatively lower costs [8]. India is an emerging centre for performing large numbers of LDLTs in its private sector, attracting patients from surrounding countries [14].

Globally, transplant programmes face major shortages of donor livers. To improve access to donor livers, there are several strategies implemented across various levels. Europe has several national and transnational organ exchange organizations supported by donation policies and legislations and standard disease severity scoring systems to select the recipients [15]. In the United Kingdom, there is a system of notification of transplant centres in the National Health Service when there is available liver anywhere in the country, through the National Organ Retrieval Service [16]. The United States, Europe, Japan, South Korea and China maintain transplant registries to facilitate access to donor livers. Similar efforts towards establishing a national registry are being undertaken in India. Among the African countries, South Africa reports liver transplantation in several liver centres. But none of the African regions have national transplant registries [16]. To overcome the low numbers of liver donations, Argentina and Paraguay have implemented the ‘hard’ presumed donor law or the Justina Law [17]. According to the Justina Law, all adults over 18 years in these countries are organ donors unless they expressly indicate otherwise before death. The purpose of this law is to balance the family's refusal of cadaveric donation.

## CHALLENGES TO LIVER TRANSPLANTATION IN LMICS

LT centres in the LMICs face major challenges with a lack of adequate facilities, organization and trainings to improve their capabilities. Across many countries, there is a lack of government support in developing organ donation legislation, establishing successful and workable organ donation networks, lack of investment in training of human resources and upgradation of hospital capabilities [14, 17, 18]. The lack of legislation regarding the use of deceased‐donor livers for transplantation limits transplant surgeries in LMICs [8].

The structure of the healthcare and funding systems, policies and expenditure per capita also play a role. Some developing countries have two‐tier healthcare systems which provide easy access to transplant services. Social, cultural and religious factors, especially in relation to cadaveric donations, are a major barrier toestablishing LT services, especially in Asian countries [19].

## LIVER TRANSPLANTATION IN SRI LANKA

Sri Lanka carried out its first successful cadaveric LT in 2010, followed by the first live‐donor transplant in 2012, the first transplant for acute liver failure in 2018 and the first successful paediatric transplant in 2020 [20, 21]. As of 2021, 22 liver transplantations have been conducted in the country [12]. In Sri Lanka, the common indications for adult LT surgery are non‐alcoholic steatohepatitis, cirrhosis or cryptogenic cirrhosis, hepatocellular carcinoma and alcohol‐associated liver disease [5]. In children, biliary atresia is the most common indication followed by hepatocellular carcinoma and Wilson disease [22]. The candidates for liver transplantation services are expected to be on the rise in the coming years as liver diseases continue to remain the third highest cause of mortality in Sri Lanka over the past decade [23].

The Transplantation of Human Tissues Act, No.: 48 of 1987 provides a policy framework for the donation of human bodies and tissues for therapeutic, scientific, educational and research purposes for removal and use on living persons [24]. In late 2010, the Ministry of Health, Sri Lanka implemented the National Organ Donor Program for deceased donor organ transplantation to strengthen the process of organ donation and transplantation [25]. The lack of adequate donor livers could be due to a lack of infrastructure development and the low number of available deceased‐donor organs [26]. Further steps to streamline and strengthen the deceased donor organ transplantations in the state health sector were taken in 2015 by appointing institutional transplant coordinators, allocating dedicated surgical theatres for organ harvesting from deceased donors for organ transplant when a need arises and arranging for logistics on transportation and consumables [27].

In 2020, the ‘National strategic plan on organ, tissue and cell transplantation of Sri Lanka, 2022–2026’ was adopted [26]. The document recognizes organ, tissue or cell transplantation as a life‐saving measure in the face of increasing disease burden requiring such therapies. The document acknowledges the need for a viable framework to allow organ donation in a manner that is socio‐culturally acceptable and enhances situations for optimal organ harvesting. It also envisions equitable access and transparency in transplantation processes with adequate protection of the health, welfare and rights of both living donors and recipients.

In Sri Lanka, LT services are provided in two geographic zones: the western zone and the central zone. The Colombo North Centre for Liver Diseases (formerly Colombo North Hepatopancreaticobiliary and Liver Transplant Unit) caters to a full range of adult and paediatric liver diseases in collaboration with the Colombo North Teaching Hospital. The centre is a dedicated multidisciplinary healthcare facility established in 2012 and is manned by specialists attached to the Faculty of Medicine, University of Kelaniya. It is the country's leading LT unit and maintains the largest database for pre‐ and post‐transplant patients. The unit is supported by the ‘REVIVE Project’ aiming to upgrade and maintain the facilities and provide financial assistance to the patients in need. In addition, the Vascular and Transplant Unit of the National Hospital of Sri Lanka, Colombo, and Sri Jayawardhanapura General Hospital are other leading centres in the western zone. In the central zone, the National Hospital in Kandy and Teaching Hospital in Peradeniya provide LT services.

Although organ transplant surgery is associated with high costs in other countries, it is provided free of charge in the state health sector in Sri Lanka. Any patient with a liver problem may be referred to the specialist gastroenterology clinic through the medical officer in the outpatient department of the government hospital or through the specialist/medical officer in general medical/surgical clinics.

## CHALLENGES IN LIVER TRANSPLANT PROGRAMME IN SRI LANKA

At present, there are not enough specialized and trained doctors to manage the two LT zones. The current teams are fully engaged in providing other therapeutic services and in providing training to new doctors. The Ministry of Health funded a Liver Transplant Training Program held in Singapore in 2019 for 14 health staff members, including consultant specialists, medical officers and nursing officers [28]. However, with the ongoing economic crisis in the country, the overall cost of LTs has skyrocketed. Although the surgery and other medical fees are free in the government sector, the out‐of‐pocket expenditure the patient and the family bear with respect to travel, food and lodging and payment for some tests and drugs have become a major barrier.

In addition, Sri Lanka also faces a major problem with low organ donors and a long waiting list. Cultural beliefs and customs, and lack of knowledge and legislation on Do Not Resuscitate orders, and the differences of the opinions or lack of knowledge of withdrawal/withholding of life‐sustaining treatments among medical professionals contribute to low volumes of organ donation [29]. To address the ethical issues surrounding end‐of‐life care, a practice guideline was adopted in 2021 with the aim to safeguard the rights and dignity of the dying person and also to provide necessary support to the relatives [30]. This guideline is also expected to influence the patient and family's decisions on organ donation while maintaining the balance of respecting the patient's wishes and ensuring adequate autonomy [28].

Where organ donation is strongly influenced by socio‐cultural beliefs, civil society organizations play an important role in public discourse surrounding medical ethics and in changing public opinion. One such organization is the Organ Donation and Transplantation Foundation (ODTF) Sri Lanka, a non‐profit organization at the Sri Jayawardhanapura General Hospital. It facilitates the national organ donor and transplant programme through mobilization of social support and funding of research on medical initiatives and implementation of deceased organ donation programme.

To improve access to donor livers in Sri Lanka, there is a need to establish a national donor registry to identify deceased and potential donors. Such a registry would also help in ensuring a transparent process in organ donation and organ transplantation procedures. In addition, the country needs to implement dedicated public health programmes and interventions to reduce the burden of preventable causes of chronic liver disease.

## CONCLUSION

Liver diseases constitute an important share of the mortality and morbidity burden among diseased adults and children. LTs can improve the survival and quality of life in those with acute and chronic liver diseases. LT programmes across many countries face a shortage of donor livers and lack funding, infrastructure and trained professionals. Sri Lanka provides LT services through its state health sector. Even though the country has appropriate organ donation legislation, obtaining donor livers is a major challenge. Although transplant surgeries are provided free of cost to the patients, the ongoing economic crisis is a major barrier to expanding the services and training transplant teams. With changing epidemiology leading to an increasing burden of liver diseases, transplant services will play a major role in patient care in the coming years.

## AUTHOR CONTRIBUTIONS

*Conceptualization; investigation; writing – original draft; writing – review and editing; resources; visualization*: M. J. S. Jayarathna. *Conceptualization; writing – review and editing; resources*: B. K. Dassanayake. *Conceptualization; writing – review and editing; supervision; resources; software; writing – original draft*: Thinley Dorji. *Writing – review and editing; supervision*: Don Eliseo Lucero‐Prisno. *Writing – review and editing; validation*: S. Samarasinghe. *Writing – review and editing; supervision; validation*: Vasanthi Pinto and M. D. Lamawansa.

## CONFLICT OF INTERESTS STATEMENT

Thinley Dorji, Don Eliseo Lucero‐Prisno III and M D Lamawansa are members of the editorial board of this journal. They were excluded and blinded from all processes related to peer review and acceptance of this article for publication.

## FUNDING INFORMATION

The authors received no funding for this study.

Global Observatory on Donation and Transplantation at https://www.transplant‐observatory.org/summary/

World Health Organization at https://www.who.int/news‐room/fact‐sheets/detail/the‐top‐10‐causes‐of‐death

Ministry of Health In: Government of Sri Lanka at http://www.health.gov.lk/

Sri Lanka Medical Association at https://slma.lk/wp‐content/uploads/2021/12/Practice‐guidelines‐in‐EoL‐care.pdf

## ETHICS STATEMENT

Ethical approval was not required.

## Data Availability

The data that support the findings of this study are openly available in:
